# Nausea and Gastric Myoelectrical Activity Are Influenced by Hormonal Contraception in Chronic Gastroduodenal Disorders

**DOI:** 10.14309/ctg.0000000000000880

**Published:** 2025-06-26

**Authors:** Alexandria H. Lim, Chris Varghese, Gabrielle Sebaratnam, Gabriel Schamberg, Stefan Calder, Armen Gharibans, Christopher N. Andrews, Charlotte Daker, Daphne Foong, Vincent Ho, Michelle R. Wise, Gregory O'Grady

**Affiliations:** 1 Department of Surgery, The University of Auckland, Auckland, New Zealand;; 2 Alimetry, Auckland, New Zealand;; 3 Division of Gastroenterology, University of Calgary, Calgary, Alberta, Canada;; 4 Department of Gastroenterology, The University of Auckland, Auckland, New Zealand;; 5 Western Sydney University, Sydney, Australia;; 6 Department of Obstetrics and Gynaecology, The University of Auckland, Auckland, New Zealand.

**Keywords:** gastroparesis, functional dyspepsia, nausea, contraception, body surface gastric mapping

## Abstract

**INTRODUCTION:**

Chronic gastroduodenal disorders are more prevalent among young women, many of whom are hormonal contraception users. We aimed to evaluate the effects of hormonal contraception on symptom severity and gastric myoelectrical activity in people with chronic gastroduodenal disorders.

**METHODS:**

This analysis was conducted on a large international cohort of patients who met Rome IV criteria for chronic nausea and vomiting syndrome or functional dyspepsia and had undergone body surface gastric mapping using Gastric Alimetry. Symptoms were continuously reported on 0–10 Likert scales using a validated symptom logging app.

**RESULTS:**

One hundred twenty-seven people were included: 43 women using hormonal contraception, 30 not using hormonal contraception, 30 postmenopausal women, and 24 men. Hormonal contraception users had higher nausea than nonusers (3.80 [interquartile range 2.00–5.42] vs 2.25 [0.20–4.43]; *P* < 0.05), particularly when using combined oral contraceptives with hormone-free intervals compared with continuous use (5.20 [4.30–6.00] vs 2.40 [1.70–3.80], *P* = 0.02). Premenopausal women were more symptomatic than postmenopausal women and men (*P* < 0.001). Principal Gastric Frequency was higher in hormonal contraception users (median 3.1 cpm vs 3.00 cpm, *P* < 0.001) and highest with progestogen-only formulations (*P* < 0.02).

**DISCUSSION:**

Women with gastroduodenal disorders on hormonal contraception experience increased nausea in comparison with nonusers, with substantial variation dependent on contraceptive type. Hormonal contraception users also demonstrated modified gastric electrophysiology. These results imply that nonhormonal contraceptive alternatives should be trialled as a means to reduce symptoms in gastroduodenal disorders.

## INTRODUCTION

Chronic gastroduodenal disorders are poorly understood and more commonly affect young female patients who suffer a disproportionate symptom burden (1–4). Hormonal contraception may be a contributing factor, which has been clearly linked with an increased risk of gastrointestinal side effects in healthy women, including nausea and bloating (5,6). The relationship between female sex hormones and gastric symptoms may provide valuable insights toward understanding mechanisms underlying chronic gastroduodenal symptoms.

Over 150 million women worldwide use synthetic mimics of progesterone and estrogen (7). Hormonal interactions in the gastrointestinal tract have been proposed to contribute to gastric disorders (8,9). Symptoms have been seen to vary with hormonal fluctuations in the menstrual cycle in patients with chronic unexplained gastroduodenal symptoms (10–12). The gastric electrical system also seems sensitive to the administration of exogenous progesterone and estrogen, inducing dysrhythmic activity (13). Hormonal contraception could induce similar effects, but until recently, it has been challenging to reliably and noninvasively assess gastric electrophysiology and gastric symptoms in relation with contraception use at scale.

Body surface gastric mapping (BSGM) is a new noninvasive diagnostic test for assessing gastric function (14–16). Validated metrics, determined by simultaneous gastric mapping and time-of-test symptom analyses, provide a standardized system to assess gastric electrophysiology (17–19). The aim of this study was therefore to evaluate the impact of hormonal contraception on nausea in patients with chronic gastroduodenal symptoms using a commercial BSGM system.

## METHODS

A total of 129 patients were evaluated from a collaborative prospective database containing participants from 3 countries (Auckland, New Zealand; Western Sydney, Australia; Calgary, Canada). Ethical approval was granted, and all patients provided informed consent.

### Study population

People aged 18 years or older were eligible for inclusion if they demonstrated a gastroduodenal symptom burden sufficient to meet the Rome IV Criteria for chronic nausea and vomiting syndrome (CNVS) or functional dyspepsia (FD), irrespective of gastric emptying status. Exclusion criteria were the following: incomplete test record, poor test quality (artifact >50%), current gastrointestinal (GI) infection, history of inflammatory bowel disease, GI malignancy, previous GI surgery, regular cannabis use, pregnancy, or metabolic/neurological disease affecting gastric function, except diabetes.

Contraceptive status was self-reported by patients on the day of testing. Premenopausal women were considered hormonal contraception users if they were on any form of contraception that administered exogenous progesterone and/or estrogen, including oral contraceptive pills, Depo-Provera, progestogen implants (depot medroxyprogesterone acetate; Jadelle), and levonorgestrel intrauterine system. Nonusers included those using barrier methods and copper intrauterine devices, or no contraception. Postmenopausal women self-reported menopause hormone therapy use. No transgender individuals were recruited in this study.

### Study procedure

Participants fasted overnight before undergoing noninvasive BSGM using a high-resolution 64-electrode array connected to a wearable reader device (Gastric Alimetry; Alimetry, Auckland, New Zealand) (15). After a 30-minute fasting recording, participants consumed an energy bar (250 kcal, 5 g fat, 45 g carbohydrate, 10 g protein, 7 g fiber) and nutrient drink (230 kcal, 230 mL) and continued recording for a further 4 hours postprandially (14). Continuous symptom monitoring was achieved using a validated application, with subjects rating nausea, bloating, upper gut pain, heartburn, stomach burn, and excessive fullness at minimum 15-minute intervals on 0–10 Likert scales (0 “no symptoms”; 10 “worst imaginable” symptoms) (17). Early satiation was recorded after meal consumption. All individual symptom scores were used to calculate the “Total Symptom Burden Score” over the test duration (17). Further details on the standard protocol are available elsewhere (15,20,21).

### Body surface gastric mapping metrics

Spectral metrics reported by the Gastric Alimetry system were the following: Principal Gastric Frequency (PGF; normative interval 2.65–3.35 cpm), body mass index (BMI)-adjusted amplitude (22–70 μV), Gastric Alimetry Rhythm Index (GA-RI; ≥0.25), and Fed:Fasted Amplitude Ratio (≥1.08). A detailed description and validation for each metric and their reference intervals are detailed in the supplementary methods and elsewhere (18,19).

### Statistical analysis

The primary outcome was the relationship between hormone contraceptive use and nausea. Secondary outcomes were relationships between different modes of contraception with gastrointestinal symptoms and gastric myoelectrical activity. All analyses were performed using R (version 4.2.3, R Foundation, Vienna, Austria). Nonparametric data are presented as the median and interquartile range (IQR). BSGM metric and symptom comparisons were made using Kruskall-Wallis and Wilcoxon signed rank tests. Age and BMI adjustments were performed using generalized multivariable linear regression models. Post hoc analyses were adjusted with Benjamini-Hochberg corrections.

## RESULTS

Patients were from New Zealand (97/129, 76%), Australia (24/129, 18%), and Canada (8/129, 6%). Women on hormonal contraception were younger than women not on hormonal contraception (25 [20–29] vs 31 [24–41], *P* = 0.007; see Supplementary Table 1, Supplementary Digital Content 1, http://links.lww.com/CTG/B326).

### Symptoms

Of the 129 participants, 90 of 129 (70%) met Rome IV criteria for both CNVS and FD, 37 of 129 (28%) for FD only, and 2 of 129 (2%) for CNVS only. Excessive fullness was the most common symptom, experienced by 114 of 129 (88%), followed by bloating (79%), upper gut pain (78%), nausea (71%), early satiation (71%), and stomach burn (49%). Belching was the most common symptom event registered during testing, experienced by 66%, followed by reflux (31%) and vomiting (10%).

**Figure 1.**
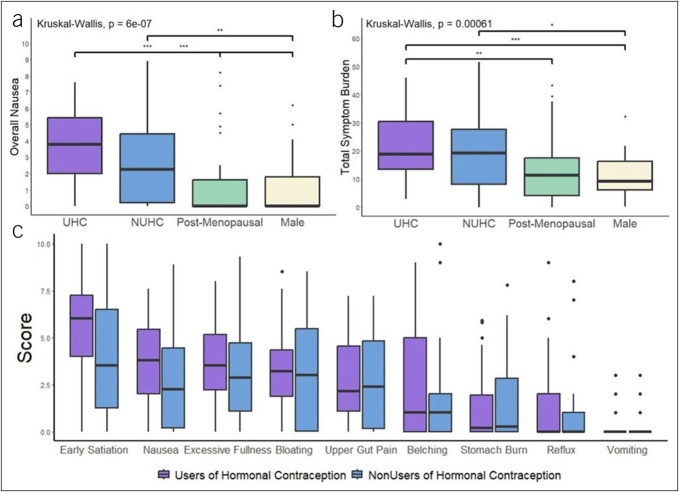
Symptom comparisons by the use of hormonal contraception. (**a**) Nausea. (**b**) Total symptom burden. (**c**) Comparison of all symptoms between hormonal contraceptive users (UHC) and nonusers (UNHC).

Nausea scores were higher in hormonal contraception users compared with nonusers (3.80 [IQR 2.00–5.42] vs 2.25 [0.20–4.43], *P* = 0.04, *P*-adjusted = 0.05), postmenopausal women (0.05 [0.00–1.65], *P* < 0.001, *P*-adjusted < 0.001), and men (0.00 [0.00–1.80], *P* < 0.001, *P*-adjusted < 0.001) (Figure 1a). Post hoc corrections for multiple comparisons revealed nausea for hormonal contraception users remained higher than nonusers of hormonal contraception (*P*-adjusted = 0.05), and both groups were higher than postmenopausal women and men (*P* < 0.02). There were no differences between hormonal contraception users and nonusers for all other symptoms (all *P* > 0.05) (Figure 1c).

As demonstrated in Figure 1b, total symptom burden scores were higher in hormonal contraception users compared with men (median 18.80 [IQR 13.53–30.43] vs 9.25 [IQR 6.18–16.28], *P* < 0.001, *P*-adjusted < 0.001). Nonusers of hormonal contraception users also experienced higher total symptom scores than men (median 19.20 [IQR 8.15–27.68], *P* = 0.03, *P*-adjusted = 0.06), but total symptom burden score did not reach significance in premenopausal women by hormonal contraceptive use (*P* = 0.14, *P*-adjusted = 0.40).

### Nausea by contraceptive type

**Figure 2.**
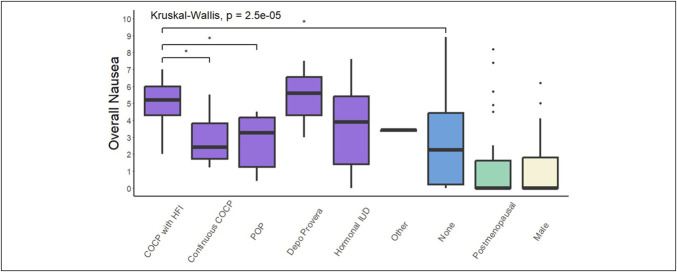
Comparing overall nausea scores with premenopausal women on hormonal contraception, stratified by type of contraception. COCP, combined oral contraceptive pill; HFI, hormone-free interval; IUD, intrauterine device; POP, progestogen-only pill.

Stratification by type of contraception revealed significant differences in nausea scores in Figure 2. Premenopausal women who used the combined oral contraceptive pill (COCP) with hormone-free intervals experienced more severe nausea than those who used the COCP continuously (without hormone-free intervals) (5.20 [4.30–6.00] vs 2.40 [1.70–3.80], *P* = 0.023, *P*-adjusted = 0.06), premenopausal women who used the progestogen-only pill (3.25 [1.23–4.15], *P* = 0.026, *P*-adjusted = 0.07), and premenopausal women who did not use any form of hormonal contraception (2.25 [0.20–4.43], *P* = 0.02, *P*-adjusted = 0.06). Users of hormonal contraception had significantly higher nausea scores than postmenopausal women and men (all *P* < 0.01). Post hoc analyses are provided in Supplementary Material (see Supplementary Digital Content 1, http://links.lww.com/CTG/B326).

Other symptoms analyzed by mode of contraceptive are summarized in Supplementary Appendix A (see Supplementary Digital Content 1, http://links.lww.com/CTG/B326).

### Overall trends

**Figure 3.**
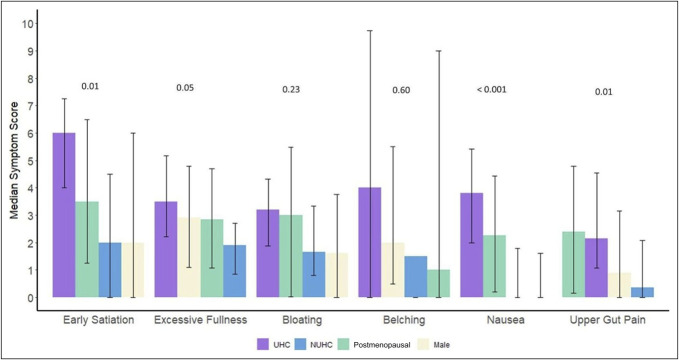
Median symptom scores by group across the 4.5-hour recording from the highest to lowest score. The median score for vomiting and reflux was 0 for all groups (not shown). NUHC, nonusers of hormonal contraception; UHC, users of hormonal contraception.

Users of hormonal contraception experienced the highest symptom burden across most of the symptoms (belching, bloating, early satiation, excessive fullness, and nausea), although not all differences reached significance. Nonusers demonstrated lower symptom scores than hormonal contraception users but higher scores than postmenopausal women and men for bloating, early satiation, and nausea (Figure 3).

### Gastric mapping metrics

**Figure 4.**
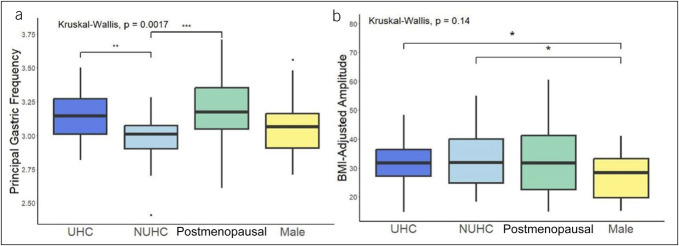
Comparative box plots for overall BSGM metrics. Refer to Supplementary Figure 1 (Supplementary Digital Content 1, http://links.lww.com/CTG/B326) for the remaining metrics. BSGM, Body Surface Gastric Mapping; NUHC, nonusers of hormonal contraception; UHC, users of hormonal contraception.

Stratification by hormonal contraception use revealed several variations within BSGM metrics (Figure 4a and b). Users of hormonal contraception demonstrated higher PGF compared with nonusers (median 3.10 cpm [IQR 3.00–3.30] vs 3.00 cpm [2.90–3.10], *P* < 0.001, *P*-adjusted = 0.004). Nonusers had higher BMI-adjusted amplitudes than men (31.90 [24.00–40.00] vs 28.30 [19.57–33.23], *P* = 0.05, *P*-adjusted = 0.15), and hormonal contraception users had higher BMI-adjusted amplitudes (33.15 [27.93–40.90], *P* = 0.01, *P*-adjusted = 0.07) and GA-RI than men (0.42 [0.32–0.56] vs 0.33 [0.26–0.43], *P* = 0.04, *P*-adjusted = 0.1). Postmenopausal women also had higher GA-RI than men (0.49 [0.32–0.58], *P* = 0.03, *P*-adjusted = 0.10). All groups demonstrated similar ff:AR (*P* = 0.39).

### PGF by mode of contraception

Variations in PGF by contraceptive type are discussed in Supplementary Appendix B (see Supplementary Digital Content 1, http://links.lww.com/CTG/B326).

## DISCUSSION

This study confirms that premenopausal women experience a substantially higher burden of symptoms than postmenopausal women and male patients. Importantly, we found that premenopausal women experience increased nausea in association with hormonal contraception use, with nausea severity being 1.7 times higher than that in nonusers. Substantial variation in nausea severity was also identified according to the specific mode of contraceptive used, with severity being 2.2 times higher for COCP users with hormone-free intervals compared with continuous users. Hormonal contraception users and postmenopausal women also showed higher gastric frequencies compared with nonusers, being highest in progesterone-specific formulations.

Gastroduodenal disorders are highly prevalent, with chronic nausea and vomiting disorders cumulatively affecting >1% of women, which is 2–3 times the rate in men (22,23). These differences are specific to premenopausal women, with a recent population-based survey of >50,000 people from >25 countries showing that CNVS was identified in 1.3% of women aged 18–39 years, decreasing to 0.7% in ages 40–64 years, and 0.4% in ages 65+ years (22). In another study on patients with gastroparesis, Parkman et al demonstrated that postmenopausal women generally experienced milder symptoms and relatively well-compensated disease compared with premenopausal women (odds ratio 0.3) (1). Our results support a possible role for female sex hormones in explaining these discrepancies (11,24,25) and implicate hormonal contraception as a plausible contributing factor.

The role of hormonal contraception in gastroduodenal disorders seems to be relatively understudied. In gastroparesis, Verrengia et al reported that nonoral contraceptive pill users had cyclical variations in symptom severity that were lacking in oral contraceptive pill users, although overall symptoms seemed similar within their limited sample (n = 20) (11). More recently, a large population-based study comprising more than 3 million patients was reported in abstract form (26). The preliminary results indicated that within 5 years of starting COCPs, patients experienced a higher risk of nausea and vomiting, epigastric pain, and bloating; higher rates of investigations with endoscopy and gastric emptying testing; and higher rates of diagnoses with gastroduodenal disorders. Although the full report of this study is still awaited, and confounding factors should be considered in a general population study, our data seem compatible.

Although our study did not specifically address causality, a causal link can be reasonably postulated between hormonal contraception and nausea because of consistent and coherent evidence across adjacent literature, including temporal and dose-response relationships, as well as biological plausibility (27). For example, nausea and vomiting are commonly observed during fluctuations or surges in female sex hormones, as seen in menstrual cycling (11,28,29), pregnancy (30), as a side-effect of initiating hormonal contraception in healthy women (31,32), and after the administration of emergency contraceptives (33). Symptoms have also been noted to fluctuate across the menstrual cycle in patients with gastroparesis, being heightened in the luteal phase (11). In addition, female sex hormones are well known to modulate several important aspects of GI function, including motility, intestinal permeability, and mucosal immunity (24), with hormonal contraception also identified as a risk factor in other GI disorders, including inflammatory bowel disease (34). Liu et al describe an increase in gastric electrophysiological abnormalities with higher doses of female sex steroids in animal studies (35). While the mechanisms of this dose-response relationship are not well established, central centers relevant to nausea expression may be involved, such as the chemoreceptive trigger zone (36).

In addition, our data demonstrated a surprising difference in nausea severity by the mode of hormonal contraception, further supporting a causal relationship (37), including the unexpected finding of a 2.2× higher nausea severity among COCP users with hormone-free intervals. Hormone-free intervals result in greater fluctuations in serum estrogen and progesterone (38), and these fluctuations in circulating hormone levels could theoretically be responsible for exacerbating nausea in patients with preexisting gastroduodenal disorders. This hypothesis is consistent with observations that fluctuations in nauseogenic stimuli can be more impactful versus when stimulus levels are stable (39). Prospective cohort studies are now desirable to confirm and extend these novel findings.

Based on the above rationale, the findings of this study are clinically significant. Chronic nausea disorders are challenging to treat. Women presenting with nausea should be asked about contraception use, and a trial of nonhormonal contraception can be considered to see if symptom improvements follow. Although healthcare professionals who prescribe contraception are aware of nausea side effects, these effects are usually evaluated only around prescription initiation; and may not be considered by gastroenterologists seeing patients with gastroduodenal disorders who are on long-term contraceptives. Changing contraceptive strategies could be a relatively underused simple intervention in GI practice, which warrants further attention. In particular, prospective studies of patients with gastroduodenal disorders could be conducted to evaluate whether switching patients taking the COCP with hormone-free intervals to a continuous COCP regime or nonhormonal methods of contraception are effective strategies.

A notable change in gastric electrophysiology was observed in association with hormonal contraception, and most importantly, elevated PGF was found in patients who had exogenous hormone delivery, particularly in progestogen formulations. This finding is consistent with a separate study recently performed by our group, which showed that similar elevations in gastric frequency occur during the luteal phase (days 16–28, with onset of menses considered as day 1) of the menstrual cycle in women not taking hormonal contraception (29). Taken together, these data likely explain why women exhibit higher overall gastric frequencies than men in normative range data sets (18). Although the changes in gastric frequency related to contraception use may seem modest, it should be noted that physiological homeostasis maintains PGF in a very tight range (2.65–3.35 cpm), and thus, a difference of 0.1 cpm reflects a relevant difference. Differences in PGF between postmenopausal women and men are currently unexplained, and while they may relate to age-associated changes in interstitial cells of Cajal (40), this requires further research. Median PGF for all groups remained within a normal range, and the differences themselves are therefore unlikely to directly induce gastroduodenal symptoms; however, they could lead to small physiological increases in gastric emptying (29). In addition, gastric dysrhythmias were not observed to be specifically associated with hormonal contraception use in this study (see Supplementary Figure 1, Supplementary Digital Content 1, http://links.lww.com/CTG/B326) (19).

Some limitations of this study are noted. The duration of contraceptive use was not available for this analysis, which may be relevant to the severity of nausea. Participants using COCP may not have been administering uniform hormonal doses, as hormonal dosing between COCP formulations is variable. The available sample size restricted subgroup analyses by hormonal type, and the novel nature of the subject limited the ability to inform a priori power calculations. Although there was an age difference between women using and not using hormonal contraceptive, these differences were accounted for in our statistical model and did not alter the significance of our primary findings regarding contraception use. We also acknowledge that the rate of CNVS-FD overlap was higher in contraception users, which is reflective of the generally overlapping nature of these symptom disorders (41). Finally, this study did not assess the role of gastric emptying as a potential contributory factor in symptom genesis, which could be important given that female sex hormones are known to modify gastric emptying (42). Future studies using combined gastric emptying and BSGM techniques would be valuable to clarify this relationship.

In summary, this study reveals novel associations between hormonal contraception use, nausea severity, and gastric electrophysiology in patients with gastroduodenal disorders, which varied by the contraceptive type used. Contraception type is an important variable to consider in patients presenting with gastroduodenal disorders, and a trial of nonhormonal contraception may be considered as a potential management strategy.

## CONFLICTS OF INTEREST

**Guarantor of the article:** Gregory O'Grady, MBChB, PhD, FRACS.

**Specific author contributions:** A.L., G. Sebaratnam, A.G., and G.O.G.: were involved in study conception and design. A.L., C.V., G. Schamberg, S.C., and A.G.: analyzed the data. A.L., C.V., G. Schamberg, S.C., A.G., and G.O.G.: were involved in data interpretation and drafting the manuscript. All authors were involved in critical revisions/final approval of the manuscript.

**Financial support:** This project was supported by the Health Research Council of New Zealand.

**Potential competing interests:** G.O.G. and A.G. hold grants and intellectual property in the field of GI electrophysiology. G.O.G., G. Sebaratnam, A.G., G. Schamberg, S.C., C.D., C.V., and C.A. are members of the University of Auckland spin-out company Alimetry. The remaining authors have no relevant conflicts to declare.

**Data availability:** Data used for analysis will be made available upon reasonable request, conditional on ethical approvals.

**Study Highlights**

**WHAT IS KNOWN**

✓ Premenopausal women are disproportionately affected by chronic gastroduodenal symptoms, particularly nausea.
✓ Sex hormones influence gastric function and symptoms, but related effects of hormonal contraception have not been well studied.

**WHAT IS NEW HERE**

✓ Women using hormonal contraception experience significantly greater nausea than nonusers, men, and postmenopausal women.
✓ Nausea severity varies by contraceptive type and is highest in users of combined oral contraceptives with hormone-free intervals.
✓ Hormonal contraceptive use is associated with elevated gastric frequencies, particularly with progestogen-only formulations.

## Supplementary Material

## ABBREVIATIONS:

BMI: body mass index
BSGM: body surface gastric mapping
COCP: combined oral contraceptive pill
CNVS: chronic nausea and vomiting syndrome
FD: functional dyspepsia
GA-RI: Gastric Alimetry Rhythm Index
GI: gastrointestinal
HFI: hormone-free interval
IQR: interquartile range
IUD: intrauterine devices
PGF: principal gastric frequency
POP: progestogen-only pill
UHC: hormonal contraceptive users
UNHC: nonhormonal contraceptive users
