# Supplementary material for: Nausea and Gastric Myoelectrical Activity Are Influenced by Hormonal Contraception in Chronic Gastroduodenal Disorders
Source: Clin Transl Gastroenterol. 2025 Jun 26;16(9):e00880. doi: 10.14309/ctg.0000000000000880 (PMC12456531; doi:10.14309/ctg.0000000000000880)
Supplement: Supplementary file 1 [file ct9-16-e00880-s001.docx]

**Supplementary Material**

**Supplementary Table 1:** Summary Baseline Characteristics

|  | | Users of Hormonal Contraception | Non-users of Hormonal Contraception | Post-Menopausal | Male |
| --- | --- | --- | --- | --- | --- |
| n (%) | | 44 | 30 | 31 | 24 |
| Age (median) | | 25 | 31 | 63 | 32 |
| BMI | | 22.4 | 23.8 | 22.5 | 23.7 |
| Diagnosis  (n, (%)) | CNVS Only | 0 (0%) | 2 (6.7%) | 0 (%) | 0 (0%) |
|  | FD Only | 1 (2.3%) | 9 (30%) | 15 (48%) | 12 (50%) |
|  | Both | 43 (98%) | 19 (63%) | 16 (52%) | 12 (50%) |
| Contraceptive Type | COCP with HFI | 9 (20%) |  |  |  |
|  | Continuous COCP | 7 (16%) |  |  |  |
|  | POP | 6 (14%) |  |  |  |
|  | Depo Provera (DMPA) | 3 (6.8%) |  |  |  |
|  | Levonorgestrel IUS | 17 (39%) |  |  |  |
|  | Other | 2 (4.5%) |  |  |  |

BMI = body mass index, CNVS = chronic nausea and vomiting syndrome, FD = functional dyspepsia, COCP = combined oral contraception pill, HFI = hormone-free interval, POP = progestogen only pill, IUS = intrauterine system

**Supplementary Appendix A**

A post-hoc analysis of nausea by contraceptive type was performed. Users of hormonal contraception demonstrated symptom differences when compared to postmenopausal women for overall bloating (3.20 [1.88 to 4.33] vs. 1.45 [0.00 to 3.58], p = 0.03, p-adjusted = 0.27), upper gut pain (2.15 [1.08 to 4.56] vs. 0.75 [0 to 2.23], p = 0.007, p-adjusted = 0.04), reflux (0.00 [0.00 to 2.00] vs. 0.00 [0.00 to 0.33], p = 0.02, p-adjusted = 0.14) and early satiation (6.00 [4.00 to 7.25] vs. 2.50 [0.00 to 6.00], p = 0.03, p-adjusted = 0.06).

Users of hormonal contraception also experienced higher symptom scores than males for upper gut pain (0.35 [0.00 to 2.08], p = 0.004, p-adjusted = 0.02), reflux (0.00 [0.00 to 0.00], p = 0.05, p-adjusted = 0.14), early satiation (2.00 [0.00 to 4.50], p = 0.002, p-adjusted = 0.01) and excessive fullness (3.50 [2.23 to 5.18] vs. 1.9 [0.85 to 2.70], p = 0.003, p-adjusted = 0.02).


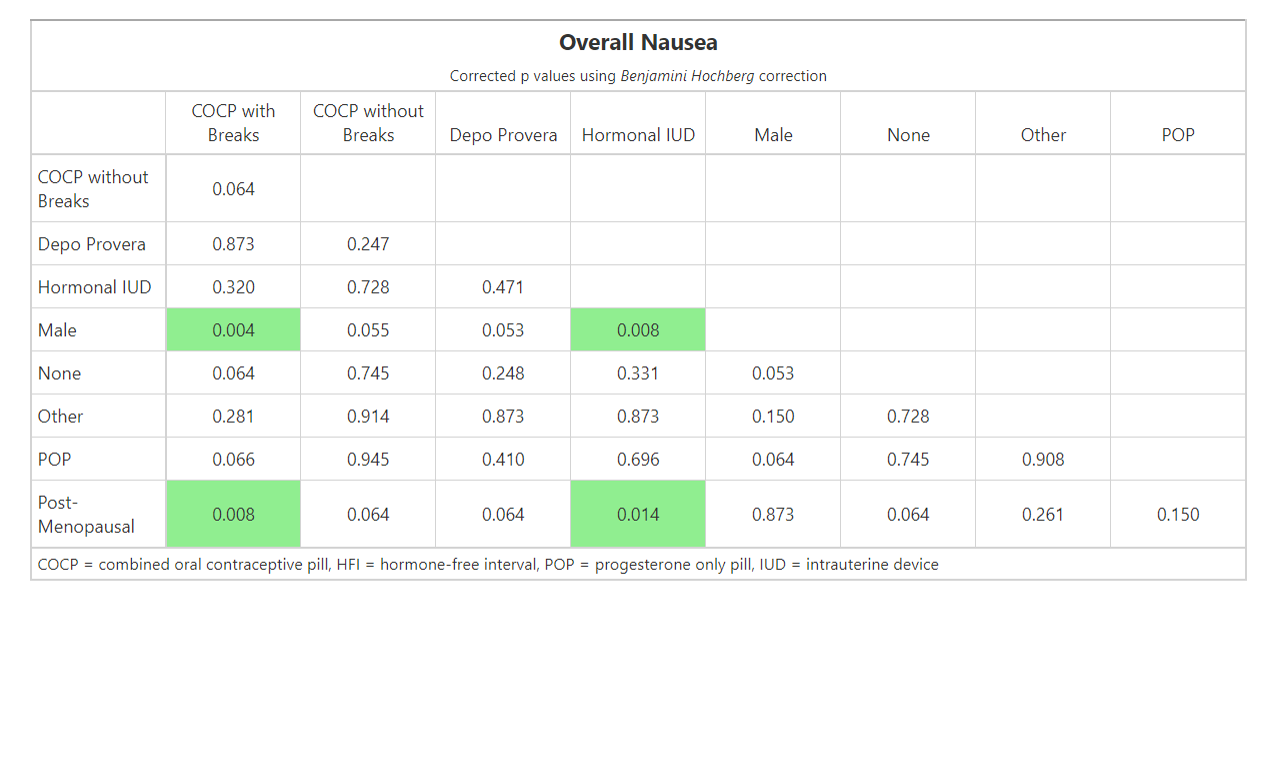


Analysis of Symptoms by Contraception Type

**
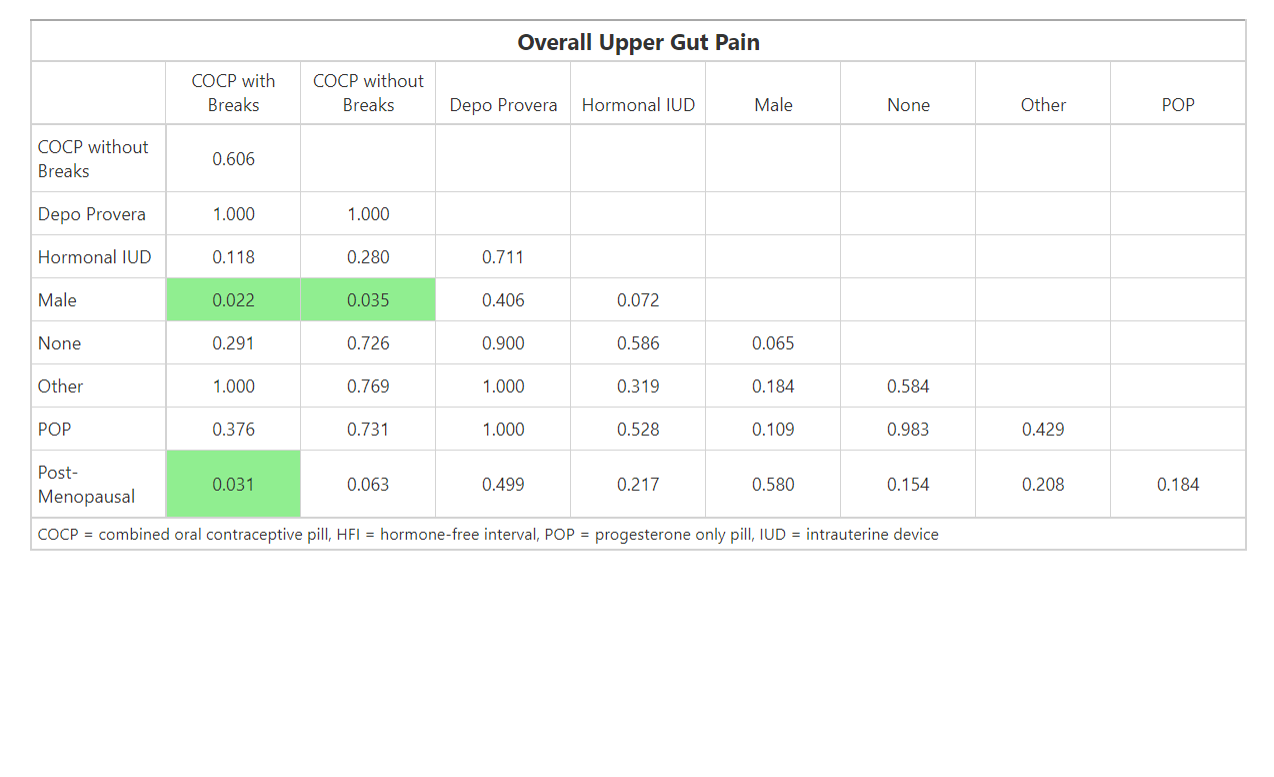
**


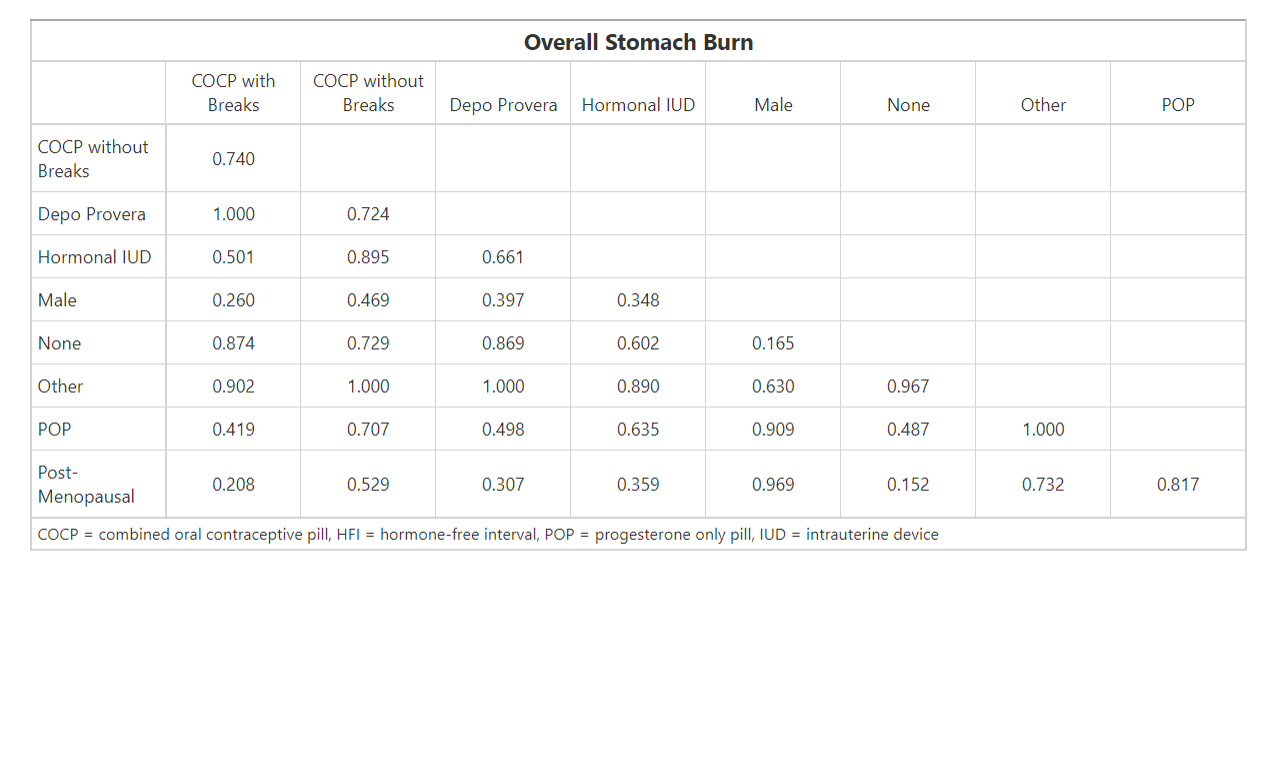

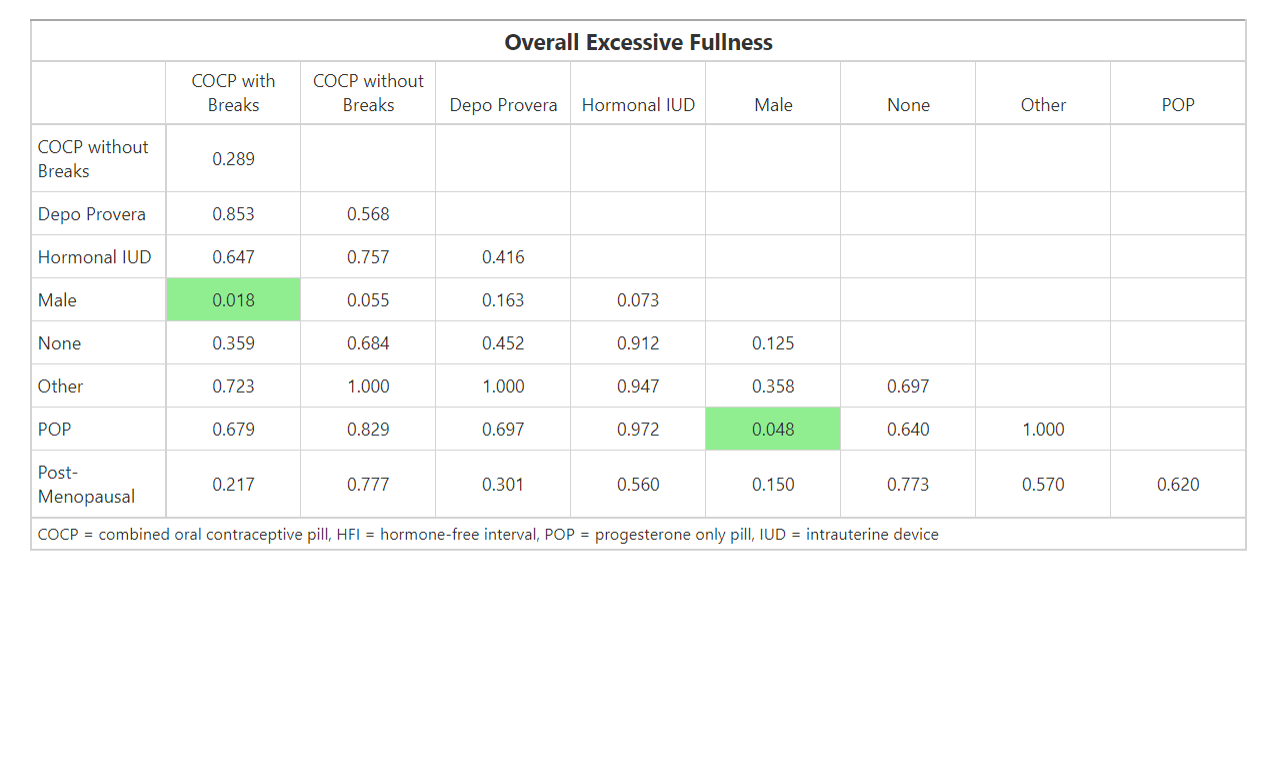

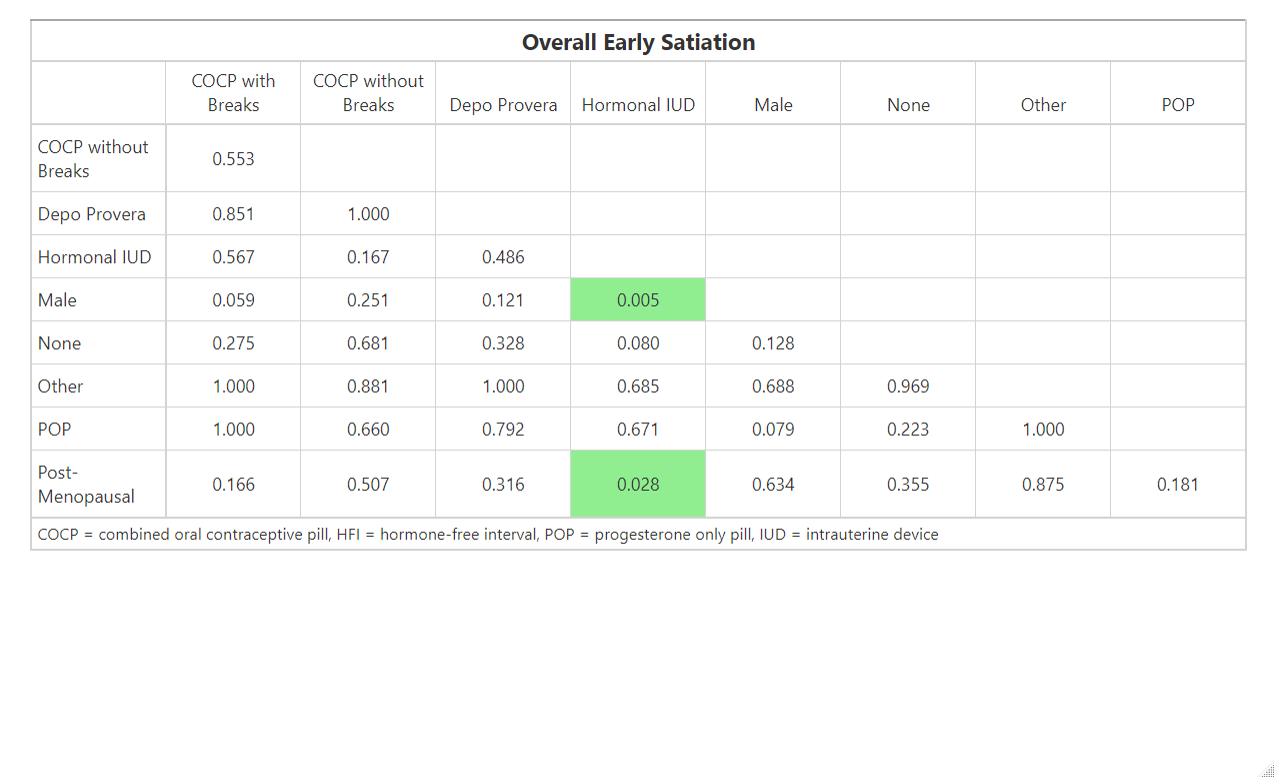

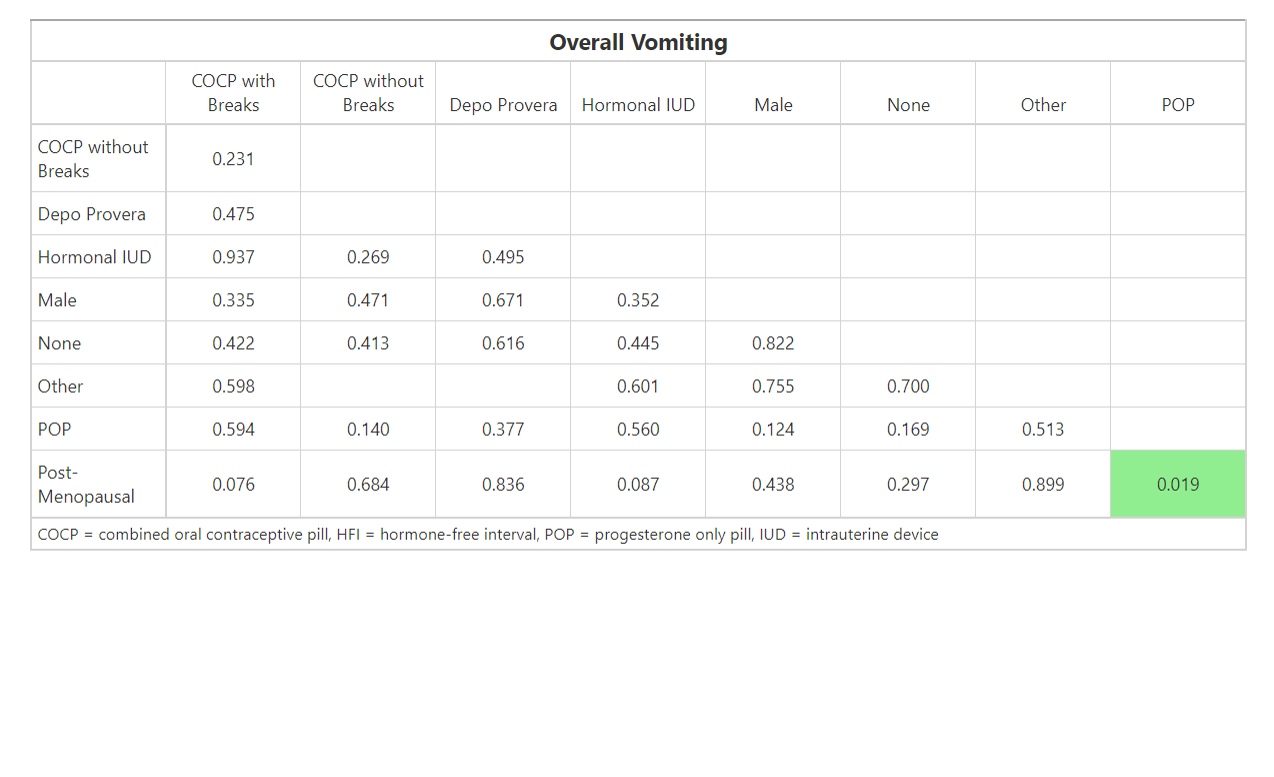

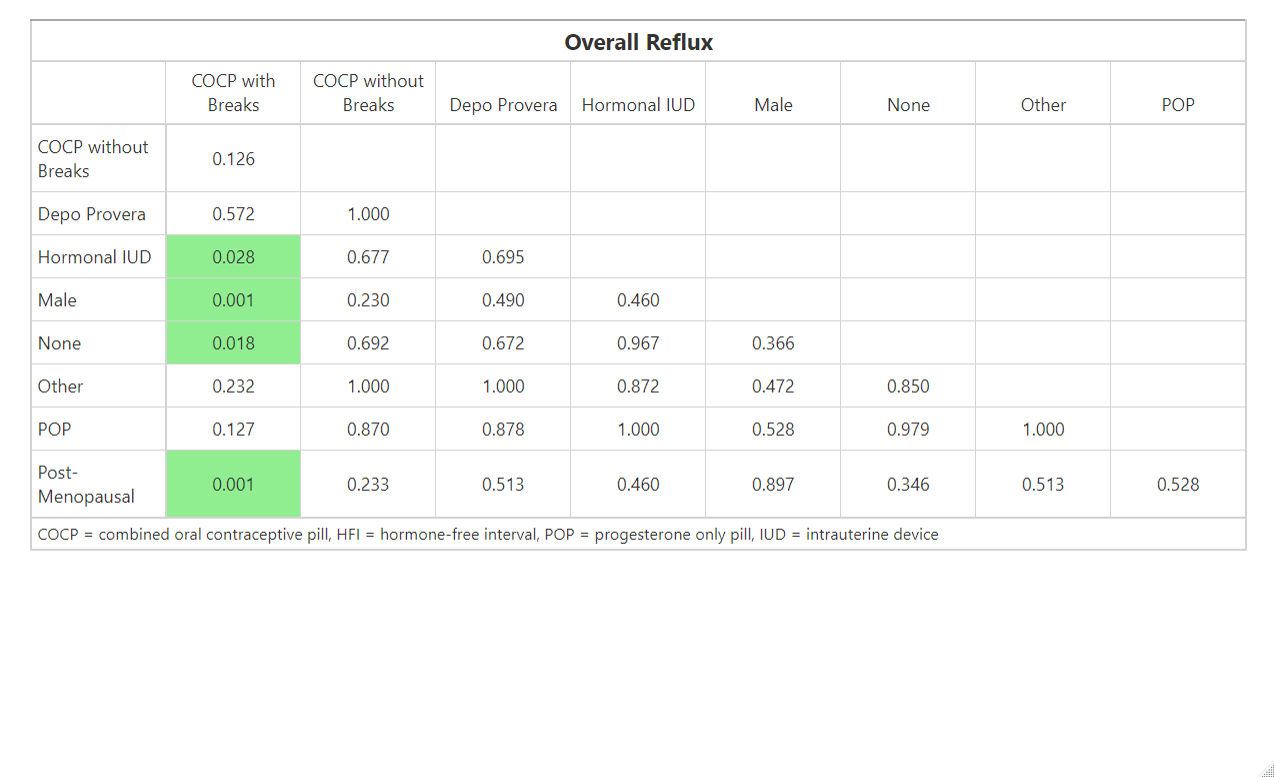

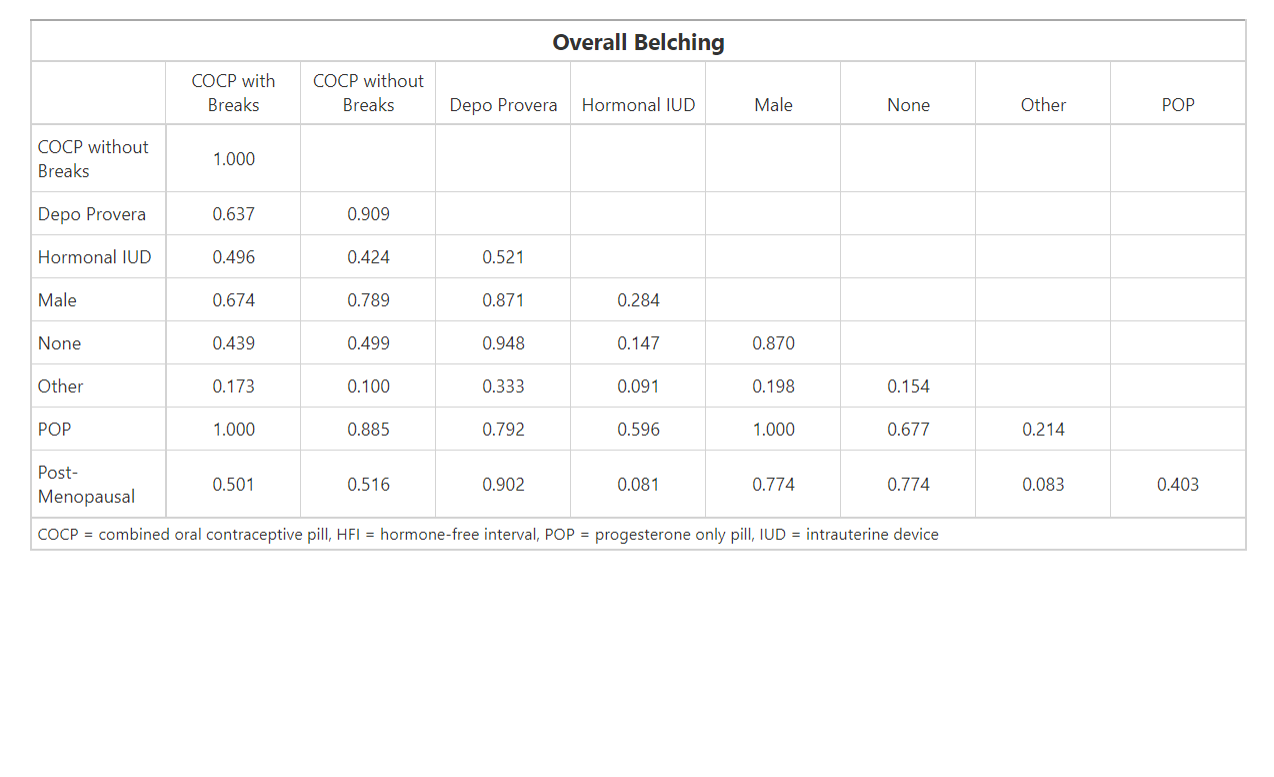


**Supplementary Appendix B**


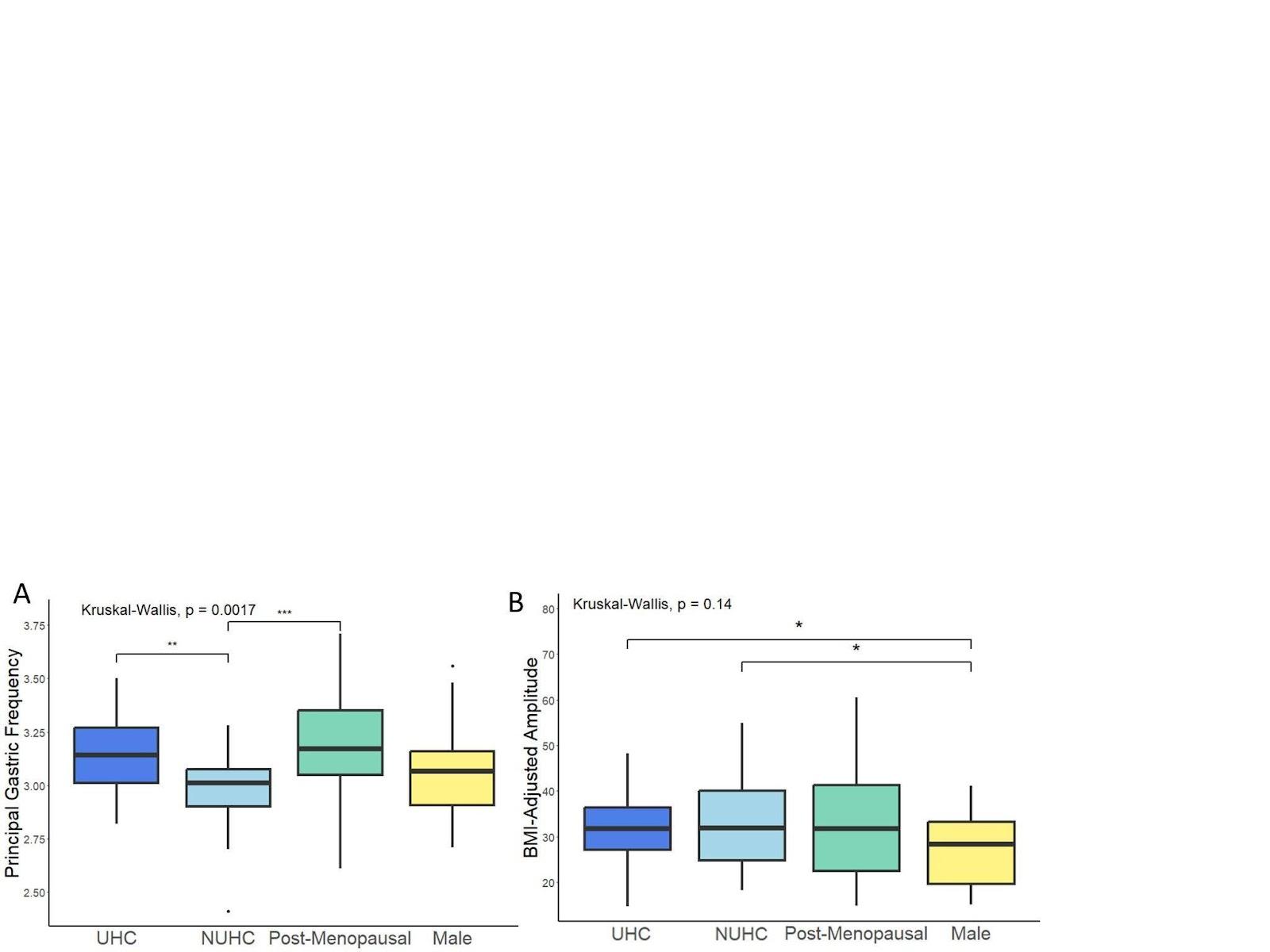


**Supplementary Figure 1:** Boxplot comparison by contraception status for remaining BSGM Metrics. A = Gastric Alimetry Rhythm Index (GA-RI), B = Fed:Fasted amplitude ratio (ff-AR).

were also observed.

Females taking the POP had significantly higher PGF (p=0.013) than females taking the COCP (regardless of hormone-free intervals) (with HFI; 3.28 [3.26 to 3.38] vs. 3.05 [3.03 to 3.07], p = 0.009), p adjusted = 0.1), (continuous; 3.14 [2.98 to 3.09], p = 0.003, p-adjusted = 0.1), females not on any form of contraception (3.01 [2.90 to 3.08], p <0.001, p-adjusted = 0.02), and males (3.07 [2.91 to 3.16], p = 0.02, p-adjusted = 0.2). Postmenopausal females also had significantly higher PGF than premenopausal females who did not use any form of hormonal contraception (3.17 [3.05- to 3.35] vs. 3.01 [2.90- to 3.08], p < 0.001, p-adjusted = 0.02) (**Supplementary Figure 2**).


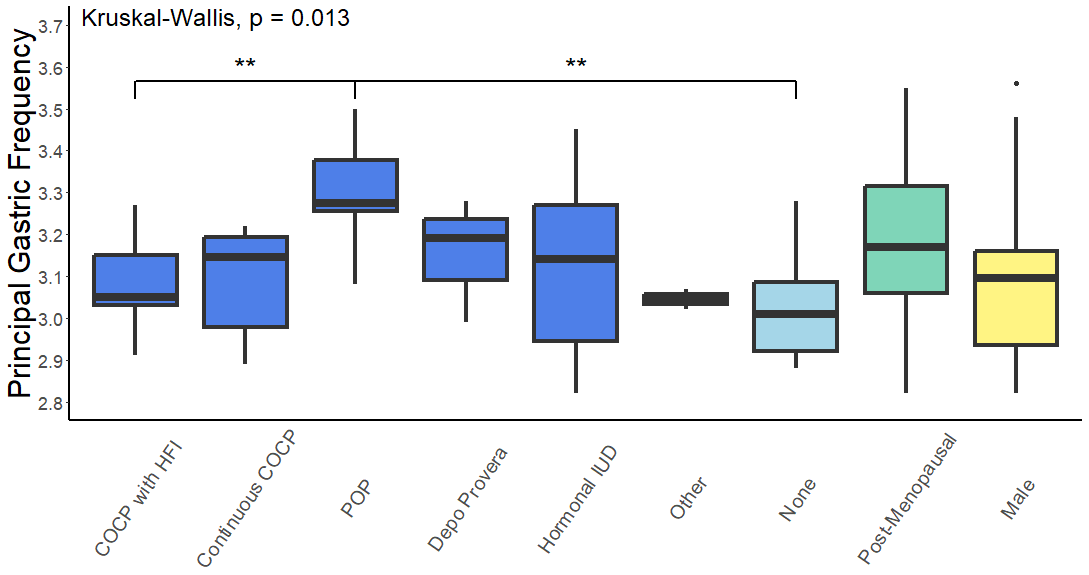


**Supplementary Figure 2:** PGF by contraception type

*Metrics Analysis - Age Adjustment*

After adjusting for age, the predicted change in odds ratio for PGF remained significant between users of hormonal contraception and non-users of hormonal contraception (exp(β): 0.84; 95% CI 0.77, 0.92; p <0.001) and revealed an additional significant difference between hormonal contraception users and males (exp(β): 0.88; 95% CI 0.79, 0.99; p = 0.03). Comparisons between non-users of hormonal contraception and postmenopausal women fell below the threshold for significance (exp(β): 1.05; 95% CI 0.91, 1.21; p = 0.5).
